# Quality appraisal of clinical guidelines for surgical site infection prevention: A systematic review

**DOI:** 10.1371/journal.pone.0203354

**Published:** 2018-09-13

**Authors:** Brigid M. Gillespie, Claudia Bull, Rachel Walker, Frances Lin, Shelley Roberts, Wendy Chaboyer

**Affiliations:** 1 School of Nursing & Midwifery, Griffith University, Gold Coast, QLD, Australia; 2 Gold Coast Hospital and Health Service, Gold Coast, QLD, Australia; 3 Optimising Health Outcomes (OHO) group, Menzies Health Institute Queensland, Griffith University, Gold Coast, QLD, Australia; 4 Division of Surgery, Princess Alexandra Hospital, Brisbane, QLD, Australia; 5 Alliance for Vascular Access Teaching and Research (AVATAR), Griffith University, Brisbane, QLD, Australia; University of Sheffield, UNITED KINGDOM

## Abstract

**Background:**

Surgical site infections (SSI) occur in up to 10% of surgeries. Wound care practices to prevent infections are guided by Clinical Practice Guidelines (CPGs), yet their contribution to improving patient outcomes relies on their quality and adoption in practice. We critically evaluated the quality of CPGs for SSI prevention during pre-, intra- and post-operative phases of care.

**Methods:**

We systematically reviewed the literature from 1990–2018 using the Cochrane Library, CINAHL, EMBASE, MEDLINE, ProQuest databases and five guidelines repositories. We extracted characteristics of each guideline using purposely-developed data collection tools. We assessed overall quality using the Appraisal of Guidelines for Research and Evaluation II (AGREE II) tool.

**Results:**

Combined searches of databases and repositories yielded 5,910 citations. Of these, we reviewed 215 full text documents. The final sample included 15 documents: 6 complete CPGs, 3 CPG updates, and 6 supplementary documents. The overall %mean scores across AGREE II domains for CPGs were: 1) *scope and purpose* (%mean ± SD = 86.3±23.5); 2) *stakeholder involvement* (%mean ± SD = 64±31.0); 3) *rigour of development* (%mean ± SD = 68.7±30.6); 4) *clarity and presentation* (%mean ± SD = 88.5±16.7); 5) *applicability* (%mean ± SD = 44±30.2); and, 5) *editorial independence* (%mean ± SD = 61±37.6). Based on individual AGREE II domains and overall scores, we appraised 4 out of 6 CPGs (inclusive of updates) as “recommended” for use in practice. Overall agreement among appraisers was excellent (ICC 0.86 [95%CI 0.73–0.94] - 0.98 [95%CI 0.96–0.99]; *p* <0.001).

**Discussion:**

International interest in CPG development has resulted in refinements to methodologies, which has led to improvements in the overall quality of the product.

**Implications for translation:**

Given the domains that received the lowest scores, it is clear that we need more consumer involvement and better consideration of the implementation challenges with CPG uptake and sustainability.

## Introduction

Over 234 million surgeries are performed around the world every year[1], yet despite the remarkable advances in surgical technologies and anaesthetic techniques, surgical site infections (SSIs) remain a major cause of patient morbidity and mortality.[2, 3] SSIs are potential complications associated with any surgical procedure; however they are the most preventable hospital acquired infection (HAI).[4] It is estimated that SSI will occur in up to 9.5% of inpatient surgical procedures.[3, 5] SSI is defined as any infection occurring within 30 days after surgery or within 12 months of surgical implantation of a prosthesis or foreign body.[5] Evidence-based wound care plays a significant role in reducing the physical, psychological, social and economic burden SSIs have on healthcare systems, patients and their families.

Decisions that health professionals make relative to wound management have important implications for patient outcomes.[6] Clinical Practice Guidelines (CPGs) offer guidance in the standardisation of care, and improve the allocation and utilisation of finite healthcare resources and reduce waste. However, the potential of CPGs to enhance wound care practice and reduce the risk of SSI is dependent on their quality, as well as uptake and adoption in practice [6]. The purpose of this systematic review was to evaluate the quality of the CPGs and strength of their recommendations that inform wound care practices in SSI prevention. Identification of the strengths and limitations of the CPGs may ultimately drive future improvements in their quality and applicability. The results of this review may also assist in the decision making of policy makers and senior clinicians relative to implementing evidence informed practices in SSI prevention.

## Methods

### Study overview

We were guided by the *Preferred Reporting Items for Systematic Reviews and Meta‑Analysis (PRISMA) statement* [7], PRISMA 2009 Checklist (S1 Checklist), and the *Cochrane Handbook for Systematic Reviews of Interventions*[8] to undertake this systematic review and report the results. As part of this process, we identified research questions *a priori* and registered the review protocol with the international prospective register of systematic reviews (PROSPERO registration number: 42017073205).

### Objectives

The objective of this systematic review was to critically appraise the *overall* quality of published guidelines for the prevention of SSI using the Appraisal of Guidelines for Research and Evaluation II (AGREE II) tool. Subsumed under this objective were the following questions:

1. Using the AGREE II tool, what is the quality of the CPGs and strength of their recommendations for the prevention of SSI?
2. Have the CPGs been revised, updated or improved over time?

As part of the appraisal process, we evaluated the similarities and differences of the main recommendations of the guidelines in parallel with the evaluation of their quality.

### Eligibility criteria

We applied the following *inclusion* criteria:

- Published international and national guidelines on the management and/or prevention of SSI;
- Published as full-text between January 1990 to February 2018;
- Guidelines published in English, as these are the most accessible and widely available;
- Most recent complete guideline (from a single working group i.e. CDC) and any partial revisions for the guideline published thereafter;
- SSI prevention/management guidelines that make recommendations across the pre-operative, intra-operative and post-operative phases; and,
- Include an explicit statement identifying the document as a ‘guideline’.

We applied the following *exclusion* criteria:

- Guidelines under development;
- Guidelines specific to one institution or surgical specialty (i.e. local hospital guidelines or orthopaedic surgery, e.g., Smith & Dahlen[9]);
- Guidelines inclusive of only one phase of care, e.g., Ubbink *et al*[10] (i.e., postoperative phase focusing on wound care and pain management)
- Complete guidelines with publication dates that have been superseded by more recent complete guidelines (i.e. the 1999 CDC guidelines have superseded the 1992 guidelines);
- Guidelines that only cover one aspect of SSI prevention (i.e. antibiotic prophylaxis); and,
- Clinical practice standards, defined as a statement reached through consensus, which clearly identifies the desired outcome. Usually used in audit as a measure of success.[11]

### Data sources and search strategy

With the assistance of a health librarian, we conducted systematic electronic searches using the Cochrane Library, Cumulative Index of Nursing and Allied Health Literature (CINAHL), EMBASE, MEDLINE and ProQuest databases. We tailored searches to the capabilities of each database, and where appropriate used either Medical Subject Headings (MeSH) or keywords, and Boolean connectors AND, OR and NOT to combine search terms. Since computerised searches identify only 50% of eligible studies,[12] ancestry searching and journal hand-searching were also conducted. We imported all database results into an Endnote (v X7, Clarivate Analytics) reference manager program prior to screening. We supplemented the database searches by searching guidelines repositories. These included *The National Guideline Clearinghouse*, *New Zealand Guidelines Group*, *The National Institute for Health and Care Excellence (NICE)*, *The National Health and Medical Research Council (NHMRC)–Australian Clinical Practice Guidelines*, *CPG Infobase*: *Clinical Practice Guidelines (Canadian Medical Association)*, *Scottish Intercollegiate Guideline Network (SIGN)*, *Clinical Key* (Elsevier), and *BMJ Best Practice*. An example of the searches undertaken across database and guideline repositories is provided in S1 Table, MEDLINE (EBSCO) search strategy and guideline repository searches.

### Data screening and extraction

Groups of titles and abstracts were assigned to three review authors who independently screened their allocated sample, and those deemed to meet the inclusion criteria were assessed further in full text. A fourth reviewer arbitrated where there was a lack of clarity around inclusion. We documented the reasons for exclusion. One review author performed data extraction using an extraction tool specifically developed for this review based on guideline characteristics relative to quality (AGREE II [13]).

### Quality assessment of CPGs

To appraise the overall quality of each included CPG, we used the Appraisal of Guidelines, Research and Evaluation (AGREE) II statement [13] to guide our systematic assessment of all published CPGs eligible for inclusion. It is generic and can be applied to guidelines in any disease area targeting any step along the healthcare continuum, including screening, diagnosis, treatment or interventions.[13] The AGREE II tool has 23 items comprising six quality-related domains (*Scope and purpose*, *Stakeholder involvement*, *Rigour of development*, *Clarity of presentation*, *Applicability* and *Editorial independence*) and a 24^th^ overall assessment item.[13] A brief description of the criteria in each of these domains is included as an S2 Table. AGREE II domain definitions. The AGREE tool was updated to AGREE II to (i) encompass a greater consideration of health generally as opposed to a specific ‘clinical’ focus; (ii) move items to more appropriate domain categories; and (iii) the addition of items (i.e. strength and limitations of the body of evidence in Domain 3: Rigour of Development).[13]

The redesigned AGREE II tool uses a 7-point Likert scale, 1 = strongly disagree through to 7 = strongly agree.[14] Scores for each domain were calculated by summing all scores of the individualised items within each domain and then standardising as follows: (obtained score minus minimal possible score) divided by (maximum possible score minus minimum possible score).[14] The minimum standardised score for each domain was 0% and the maximum was 100%. Scores were converted to percentages values for %mean score determination. The scale was transformed to the following: 1 = 14.29%, 2 = 28.57%, 3 = 42.86%, 4 = 57.14%, 5 = 71.43%, 6 = 85.71%, and 7 = 100%. A guideline is ‘‘ recommended” if most of the domains (≥ 4) scored above 50%. A guideline is ‘‘recommended with modifications” if 3 domain items scored above 50%. A guideline is ‘‘not recommended” if ≥4 domains scoring less than 50%. Appraisers assigned scores depending on the completeness and quality of reporting and scores increased as more criteria were met.

CPGs deemed eligible for inclusion were appraised independently by members of the authorial team using the AGREE II tool.[13] Each CPG was assigned three appraisers. The AGREE II requires at least two appraisers to reach acceptable interrater agreement on the tool.[14] We classified the degree of agreement according to the scale by Landis and Koch;[15] poor (0.00), slight (0.00 and 0.20), fair (0.21 to 0.40), moderate (0.41 to 0.60), substantial (from 0.61 to 0.80), and very good or almost perfect (0.81 to 1.00). Prior to completing appraisals of each CPG, all review authors completed the AGREE II Online Training Tool [16] and participated in three rounds of calibration. Authors completed online appraisals using the My AGREE PLUS platform.[17] During the quality appraisal process, we met regularly to discuss results, clarify information and resolve differences by consensus. We measured the mean proportion of agreement relative to overall quality (AGREE II) of included CPGs among three assessors in each AGREE domain using the ICC with 95% confidence intervals (CI).

## Results

**Fig 1:**
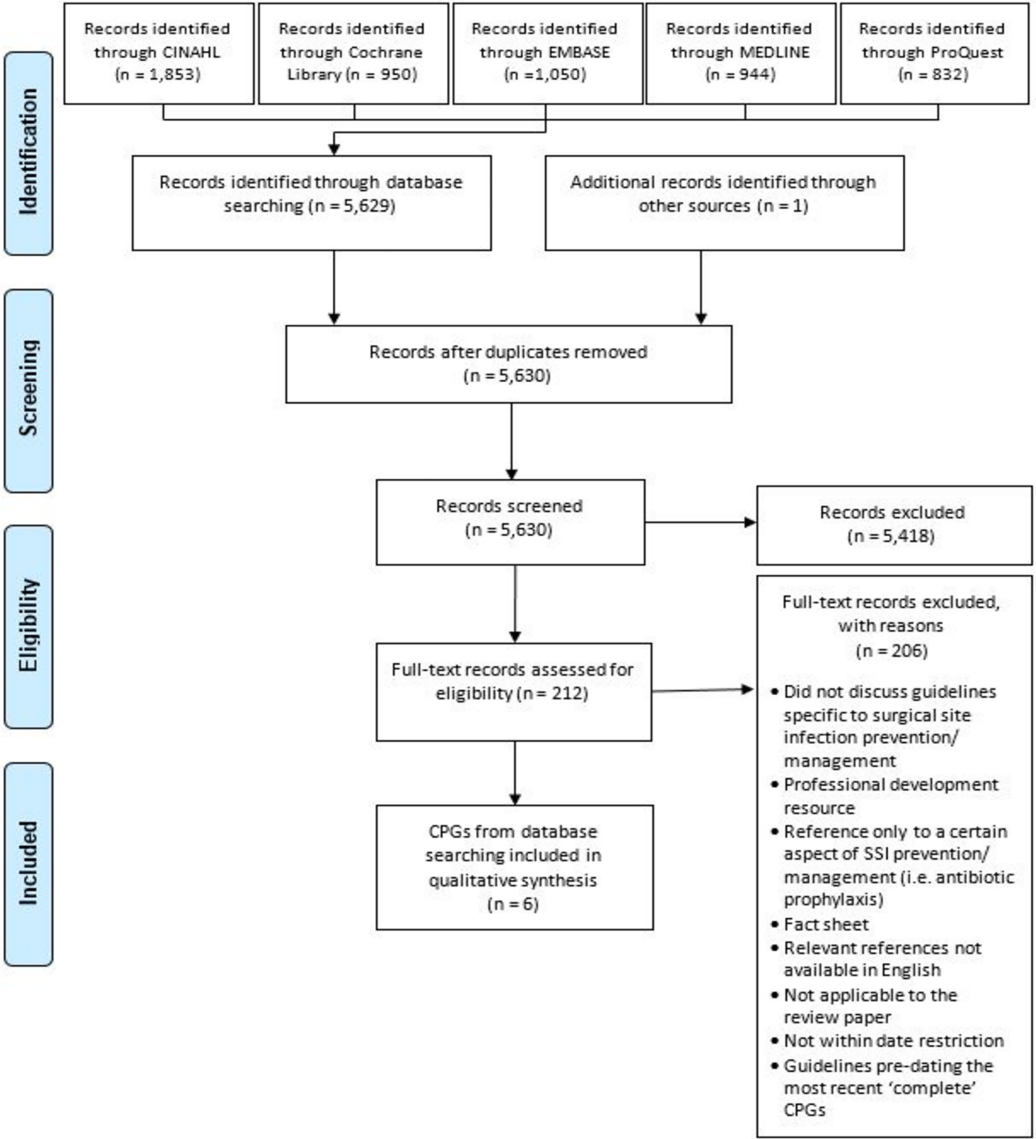
Search strategy for library databases (final search undertaken on 02/03/2018).

**Fig 2:**
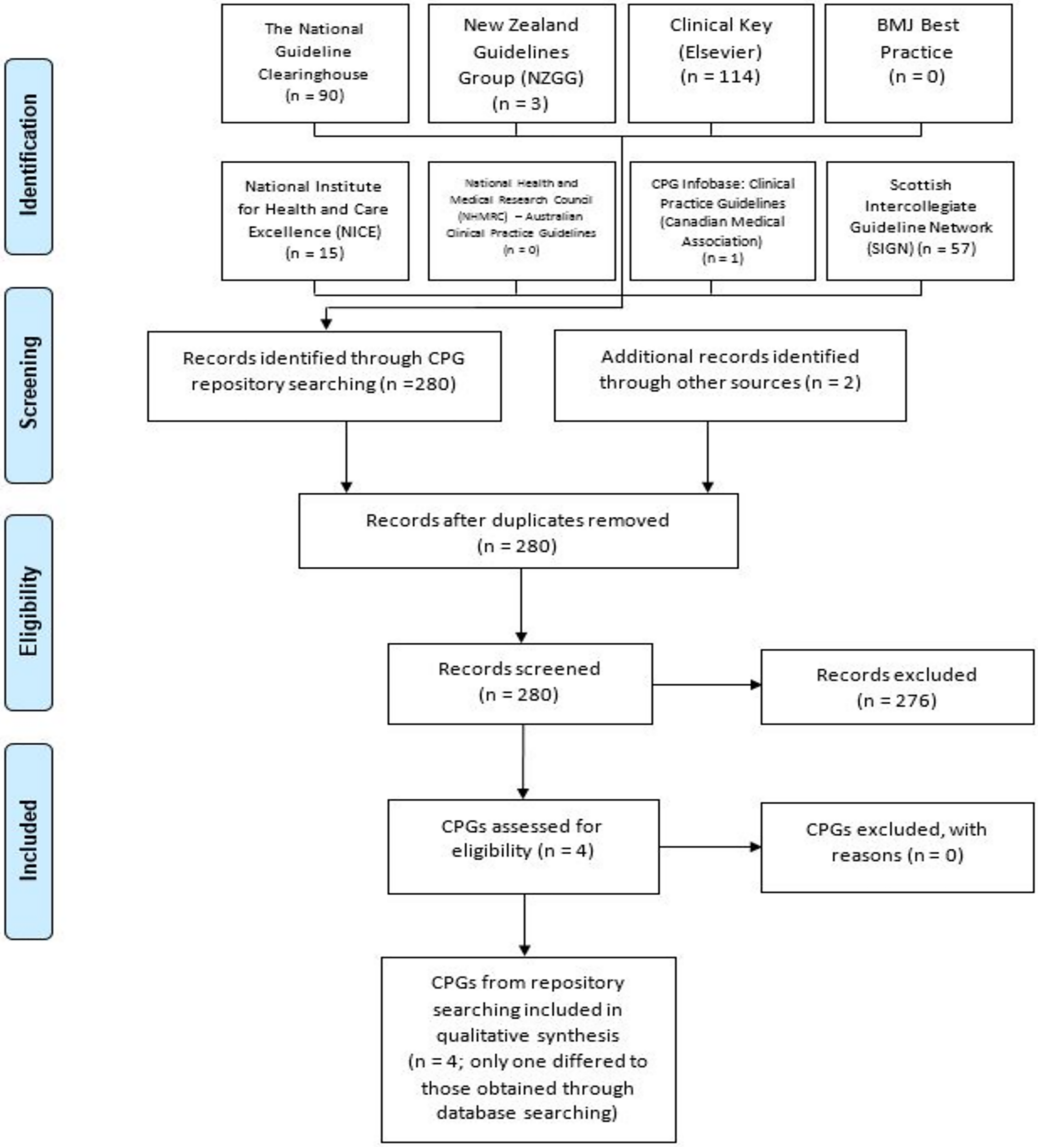
Search strategy for guideline repositories (final search undertaken on 02/03/2018).

Our electronic database searches retrieved 5,630 documents; of these, we considered 212 for full text screening. Among these, we excluded 206 documents. Using guidelines repositories, we identified 280 documents, and of these, excluded 276 documents. In total, we included 15 documents in the final analysis. The total number of guidelines included six complete CPGs,[5, 18–22] 3 updates [18, 23–25] and six additional documents (including a supplement)[25–29] that supplied information not present in the original or update CPG documents relevant for appraisal. Figs 1 and 2 illustrate the complete selection process based on the database and guidelines repositories using PRISMA [7] flow charts. Table 1 details the characteristics of the six CPGs and their included updates. The included CPGs and their updates were published between 1999 and 2018. Of the six complete CPGs, 3 [5, 18, 19] were developed in the United States. Refer to S3 Table–Summary of sources where CPGs were obtained.

**Table 1:** Characteristics of CPGs regarding SSI prevention.

	CDC 1999 & 2017 update	NICE 2008 & 2014 update	WHO 2016	Strategies to Prevent SSI 2008 & 2014 update	Bulletin of the American College of Surgeons, 2016	University of Toronto 2017
**Original CPG title**	Guideline for Prevention of Surgical Site Infection, 1999	Surgical site infection prevention and treatment of surgical site infection	Global Guidelines for the Prevention of Surgical Site Infection	Strategies to prevent surgical site infections in acute care hospitals	Guideline for prevention of surgical site infection	Surgical Site Infection Prevention: A Clinical Practice Guidelines developed by the University of Toronto’s Best Practice in Surgery in collaboration with the Antimicrobial Stewardship Program
**Date published**	1999/2017	2008/2014	2016	2008/2014	2016	2017
**Country of origin**	US	UK	Switzerland	US	US	Canada
**Objective of CPG**	Provide recommendations for the detection and prevention of SSI.	Provide guidance on the patient’s journey through out the pre/intra, & postoperative phases of care	Provide comprehensive evidence-based recommendations for interventions, applied during the pre/intra, & postoperative phases of care	Provide comprehensive evidence-based recommendation for detecting HAI infections.	Not stated	To make recommendations for interventions which decrease the risk of surgical site infections in surgical patients.
**Methods used to collect/select the evidence**	1999-not stated; 2017-targeted systematic review using 4 databases	2008-systematic literature reviews using 7 databases; 2014-searches based on clinical questions	Identify critical priorities using PICO; Systematic reviews of topic areas	Not stated	Not stated	Primary literature review; consideration of the WHO 2016 Global Guidelines for the Prevention of surgical Site Infection, American Society of Health-System Pharmacists (ASHP) recommendations, National Institute for Health and Care Excellence (NICE) guidelines and Canadian Patient Safety Institute (CPSI) Surgical Site Infection: Getting Started Kit.
**Methods used to analyse the evidence**	Hierarchical system used to grade levels of evidence	Hierarchical system used to grade levels of evidence	Assessment & synthesis of evidence; Formulate recommendations & dissemination	Not stated	Not stated	Not stated
**Ranking scheme to determine strength of the evidence & recommendation**	1999: 1A, 1B, 2 & no recommendation; 2017: modified GRADE system	1++, 1+, 1-, 2++, 2+, 2-, 3 & 4	GRADE system: High, moderate, low & very low	2008: Adapted from the Canadian Task Force; A I-III, B I-III & C I-III; 2014: GRADE System I-high, II-moderate & III-low	1A, 1B, 2 & no recommendation	GRADE system: High, moderate, low & very low
**Methods used to formulate the recommendations**	Expert consensus	Expert consensus	Expert consensus	Not stated	Not stated	Recommendations were tailored for practice at the University of Toronto affiliated hospitals in collaboration with the Antimicrobial Stewardship Program.
**Number of recommendations in each CPG**	72 44	59 20	33	26 28	44	21
**Method of CPG validation**	External and internal peer review	External and internal peer review	External and internal peer review	Not stated	External and internal peer review	External^b^ and internal peer review
**Intended users**	Surgeons, operating room nurses, postoperative inpatient and clinical nurses; infection control professionals; anaesthesiologists; healthcare epidemiologists; and other personnel directly responsible for the prevention of nosocomial infections.	2008-health professionals involved in the care of surgical patients; 2014-decision makers, surgical patients, their families and caregivers	Surgical team including surgeons, nurses, technicians, anaesthetists & bedside clinicians; decision makers; senior managers & infection control professionals	Acute care hospitals	Not stated	Surgeons, surgical residents and fellows; anaesthesiologists; pharmacists; and nurses caring for surgical patients
**Composition of CPG working group**	12 health professionals from infectious diseases, surgery & nursing,	2 surgeons, tissue viability nurse, theatre nurse, 2 microbiologists, surveillance coordinator, infection control specialist, 2 patient/carer representatives	4 groups: 1.Steering group 2. Guidelines development group 3. Systematic reviews group 4. External peer review group	Not stated	Not stated	7 members of the University of Toronto’s Best Practice in Surgery group in collaboration with the Antimicrobial Stewardship Program
**Number of documents included in appraisal**	3 1999 CPG (38 pages); 2017 update (8 pages); 2017 supplementary online content (600 pages)	2 2008 CPG (168 pages); 2014 update (28 pages)	1 2016 CPG (168 pages)	6 2008 CPG (11 pages); 2008 executive summary (10 pages); 2008 introduction (9 pages); 2014 CPG (23 pages); 2014 compendium (25 pages); 2014 introduction (5 pages)	2 2017 CPG (16 pages); 2017 executive summary (4 pages)	1 2017 CPG (27 pages)
**Where primary CPG can be found**	Available through CDC Stacks Public Health Publications Website: https://stacks.cdc.gov/view/cdc/7160	Available through NICE Website: https://www.nice.org.uk/guidance/cg74	Available through WHO Website: http://www.who.int/gpsc/ssi-prevention-guidelines/en/	^a^2014 update available through EMBASE Website: https://www.embase.com/search/results?subaction=viewrecord&from=export&id=L373762398	Available through Mary Ann Liebert Online Website: http://online.liebertpub.com.libraryproxy.griffith.edu.au/doi/pdf/10.1089/sur.2016.214	Available through Google search: http://bestpracticeinsurgery.ca/wp-content/uploads/2017/11/SSI-BPS-CPG-Nov20.pdf

CPG, clinical practice guideline; NICE, National Institute for Clinical Excellence; WHO, World Health Organisation; US, United States; UK, United Kingdom; SSI, surgical site infection; PICO, population intervention comparison outcome; ASHP, American Society of Health-System Pharmacists; CPSI, Canadian Patient Safety Institute; GRADE, Grades of Recommendation Assessment, Development and Evaluation; CDC, Centre for Disease Control.

^a^2008 CPG not available online; was requested through Griffith University; refer to S3 Table for details of where to obtain all documents included in appraisal.

^b^External validation information is not publically available; multiple requests for this information by the authorial team were not responded to by the working group.

### Quality appraisal

Three assessors appraised each CPG. The %mean scores for each AGREE II domain were calculated by summing the scores of the individual items in domains and then standardising minimum/maximum scores ranging from 0% to 100%. The results were as follows: 1) *scope and purpose* (%mean ± SD = 86.3±23.5); 2) *stakeholder involvement* (%mean ± SD = 64±31.0); 3) *rigour of development* (%mean ± SD = 68.7±30.6); 4) *clarity and presentation* (%mean ± SD = 88.5±16.7); 5) *applicability* (%mean ± SD = 44±30.2); and, 5) *editorial independence* (%mean ± SD = 61±37.6).

The overall quality scores of each guideline (including updates) across each domain of the AGREE II are presented in Table 2. Where CPGs included updates, we appraised the update as part of the original guideline. In the first AGREE II domain, *Scope and Purpose*, quality scores ranging from 39% (±14.3) to 100% (±0) with 5 of 6 CPGs scores over 50%. The second domain *Stakeholder Involvement*, mean scores across CPGs varied from 15% (±13.3) to 100% (±0), with 4 of 6 of CPGs with scores greater than 50%. In the third AGREE II domain, *Rigour of Development*, mean scores ranging from 21% (±20.5) to 97% (±6.9) with 4 of 6 CPGs scored over 50%. The fourth domain, *Clarity of Presentation* mean scores varied from 56% (±21.6) to 100% (±0) and all CPGs scored over 50%. In the fifth domain, *Applicability*, mean scores were much lower overall, ranging from 4% (±6.5) to 86% (±13.4), and only 3 of 6 CPGs had scores greater than 50%. In the sixth domain, *Editorial Independence*, 4 of 6 CPGs scored over 50%, with scores ranging from 11% (±11.7) to 100% (±0). Based on the appraisal of individual AGREE II domains and overall scores, 4 of 6 of the included CPGs were rated as “recommended”.

**Table 2:** AGREE II scaled domain scores of CPGs for SSI prevention.

AGREE II Domain	CDC 1999 & 2017 update	NICE 2008 & 2014 update	WHO 2016	Strategies to Prevent SSI 2008 and 2014 update	Bulletin of the American College of Surgeons, 2016	University of Toronto 2017
	% (SD)	% (SD)	% (SD)	% (SD)	% (SD)	% (SD)
**1 –Scope and Purpose**	98% (±4.8)	98% (±4.8)	100% (±0)	91% (±7.5)	39% (±14.3)	92% (±10.4)
**2 –Stakeholder Involvement**	76% (±27.8)	100% (±0)	87% (±13.8)	63% (±40.9)	15% (±13.3)	43% (±34.3)
**3 –Rigour of Development**	88% (±19.5)	97% (±6.9)	95% (±6.4)	65% (±24)	21% (±20.5)	46% (±28.3)
**4 –Clarity of Presentation**	96% (±6.3)	100% (±0)	100% (±0)	87% (±13.9)	56% (±21.6)	92% (±7.6)
**5 –Applicability**	25% (±11.3)	51% (±29.5)	86% (±13.4)	68% (±31.9)	4% (±6.5)	30% (±16.6)
**6 –Editorial Independence**	83% (±12.8)	83% (±12.8)	100% (±0)	72% (±19.5)	11% (±11.7)	17% (±5.8)
**Recommended use of this CPG**	Yes	Yes	Yes	Yes	No	No
**ICC (including overall CPG score)**	0.981	0.880	0.863	0.968	0.879	0.908

ICC, inter-class correlation; SD, standard deviation; CPG, clinical practice guideline; CDC, Centre for Disease Control; NICE, National Institute for Clinical Excellence; WHO, World Health Organisation.

Across the AGREE II domains, ICC coefficients among appraisers ranged from 0.86 (95%CI 0.73–0.94) to 0.98 (95%CI 0.96–0.99; *p*<0.001), indicating an almost perfect level of agreement.[15]

### Levels of evidence used in CPGs to inform recommendations

Table 3 shows the levels of evidence for recommendations across each of the three phases of surgical care, i.e., pre-operative, intra-operative and post-operative. Two of the recently developed complete guideline,[21, 22] and recent updates of two others [23, 30] used the GRADE system to rank recommendations. Only one CPG [25] was developed using a working group (based on expert opinion). Comparatively, there was consistent agreement in the ranking of recommendations across CPGs (including updates) relative to the following SSI prevention interventions; hair removal, antibiotic prophylaxis, and the wearing of surgical attire. However, across most other SSI prevention interventions/strategies in the preoperative and intraoperative period, agreement relative to level of evidence and the number of recommendations was inconsistent. S4 Table–Evidence level systems used across CPGs details the different evidence systems used for each recommendation identified in each CPG. S5 Table–Recommendations across all CPGs that informed Table 3 details specific recommendations across each CPG. The 2017 CDC guideline [23] included content specific to prosthetic joint arthroplasty. There were far fewer recommendations identified across included CPGs in relation to the post-operative phase, particularly pertaining to wound care.

**Table 3:** Levels of evidence for recommendations for SSI prevention as reported in included CPGs.

**Recommendations**^a^	**CDC 1999 & 2017**^b^ **update**	**NICE 2008 & 2014 update**	**WHO 2016**	**Strategies to Prevent SSI 2008 & 2014**	**Bulletin of the American College of Surgeons 2016**	**University of Toronto 2017**
**Pre-operative phase**						
**1. Showering/bathing**	1B 1B, NR	1+ NFR	Moderate	— —	WG	WG
**2. Hair removal**	1A —	1+ NFR	Moderate	A-I —	WG	Moderate
**3. Antibiotic prophylaxis**	1A-1B 1A, 1B, NR	1+, 1- —	Low–Moderate	A-I, A-II, B-II I, II	WG	Very low–High
**4. Nasal decontamination**	NR —	1+ —	Moderate	— —	WG	—
**5. Mechanical bowel preparation**	1A —	1+ —	Moderate	— —	WG	—
**6. Surgical site antimicrobial skin preparation**	1B-2 —	— —	Very low–Moderate	A-II I	WG	Low–Moderate
**7. Patient theatre attire**	— —	4 —	—	— —	—	—
**Pre-operative and/or Intra-operative phase**						
**1. *Patient Homeostasis*:**						
• **Glycaemic control**	1B 1A, NR	1- —	Low	A-II I, II	WG	—
• **Enhanced nutritional support**	NR —	— —	Very low	A-I I	—	—
• **Pre-warming**	— —	— —	—	— —	WG	Very low–Moderate; WG
• **Oxygenation**	NR 1A, NR	1+, 1- NFR	Moderate	— I	—	—
• **Discontinuation of immune-suppressants**	NR — Ortho: 1A, NR	— —	Very low	C-II III	—	—
**Intra-operative phase**						
**1. Optimal timing for antibiotics**	1A 1B, NR	2+ —		A-I II	—	Very low–Moderate
**2. Surgical attire, incisor drapes and gowns**	1B, NR 2 Ortho: NR	1+, 4 NFR	Very low–Moderate	— I, III	WG	—
**3. Disposable versus reusable drapes and gowns**	— —	— —	—	— —	—	—
**4. Surgical scrub/hand antisepsis**	1B-NR —	1+, 4 —	—	A-II II, III	WG	—
**5. Surgical site antiseptic skin asepsis**	1A, 1B 1A, 2, NR	1+, 1- NFR	—	A-II I	—	—
**6. Diathermy versus scalpel for surgical incision**	— —	1+ NFR	—	A-III III	—	—
**7. Incisional wound irrigation & intra-cavity lavage**	1B 2, NR	1+, 1- —	Very low–Low	— II	WG	—
**8. Antiseptic/antimicrobial agents prior to wound closure**	— 1B, NR	1+ NFR	—	— —	—	—
**9. Closure methods**					—	—
• **Antimicrobial sutures**	— 2	— —	Moderate	— II	WG	Moderate
• **Suture glue**	— —	— —	—	— —	—	—
**10. Selection of wound dressings** • **Prophylactic NPWT**	— —	— —	Low	— —	—	—
**11. Room traffic & ventilation**	1B, 2 —	4 —	Very low–Low	B-II, C-I III	—	—
**12. Decontamination of:**						
• **Environment & medical devices**	1B-NR —	— —	—	A-III, B-III —	—	—
• **Surgical instruments**	1B —	— —	—	B-I II	WG	—
**13. Prevention of biofilm**	— — Ortho: NR	— —	—	— —	—	—
**Post-operative phase**						
**1. Surgical antibiotic prophylaxis prolongation**	— 1A Ortho: 1A	— —	Moderate	A-I II	WG	Moderate–High
**2. Timing of dressing changes**	1B, NR —	— —	—	— —	WG	—
**3. Postoperative wound cleansing**	— —	1+, 4 —	—	— —	WG	—
**4. Topical antimicrobial agents for wound healing by primary intention**	— —	1+ —	—	— —	WG	—
**5. Dressings for wound healing by secondary intention**	— —	1- —	—	— —	—	—
**6. Wound debridement**	— —	1- —	—	A-III —	—	—
**7. Use of advanced dressings**	— —	— —	Low	— —	—	—
**8. Wound related analgesia**	— —	— —	—	— —	—	—
**9. Specialist wound care services**	— —	4 —	—	— —	—	—
**10. Antibiotic treatment of SSI & treatment failure**	— —	4 —	—	— —	—	—
**Documentation**						
**1. Surgical wound classification**	1B, 2 —	— —	—	— —	—	—
**2. Record variables associated with increased SSI risk**	1B —	— —	—	— —	—	—
**3. Calculation of operation-specific SSI rates**	1B —	— —	—	A-II II, III	—	—
**4. Report SSI rates to surgical team members**	1B —	— —	—	A-II II	—	—
**Patient/family education**						
**1. Incision care/management**	2 —	— —	—	— —	—	—
**2. Symptoms/recognising/reporting SSI**	2 —	— —	—	A-III III	—	—
**3. Smoking cessation**	1B —	— —	—	A-II I	WG	— —
**4. Discharge planning**	— —	1+, NR —	—	— —	—	—

Not reported,—; No recommendation/unresolved issue, NR; No further recommendation, NFR; Working group expert opinion, WG; CPG, clinical practice guideline; NICE, National Institute for Clinical Excellence; WHO, World Health Organisation; SSI, surgical site infection; NPWT, negative pressure wound therapy.

^a^Refer to S4 Table for an explanation of the different evidence levels and S5 Table for the recommendation from each CPG that informed Table 3.

^b^The 2017 CDC updated CPGs have a heavy focus on prosthetic joint arthroplasty (PJA); specifically, recommendations 11.A – 20.D. We have identified these recommendations in the CDC column by labelling them ‘Ortho’.

## Discussion

This is the first systematic evaluation of the quality of SSI prevention guidelines to our knowledge. Generally, the quality of these guidelines was acceptable with most evaluated as “recommended”. A “good” guideline should be scientifically valid, practical, consistent and should ultimately improve the outcomes of patients.[31] Of the CPGs identified in 235 studies assessing the effectiveness and efficiency of dissemination and implementation strategies, only 3% of the guidelines were based on good evidence.[32] Our review identified an overall improvement in the quality of the SSI prevention guidelines over time, albeit that some of the main recommendations are based on weak/low grade or inconclusive evidence. Up to 30% of all medical care adds no value to patients, and may in fact lead to harm.[33] Despite this, there are many interventions that are based on questionable evidence, and their inclusion in CPGs has been labelled an “illusionary attempt to embrace the entire clinical reality.”[34] Clearly, including small trials reporting weak evidence in CPGs have a bearing on the quality and strength of the recommendations identified.

Recommendations across the reviewed CPGs were reasonably consistent for three SSI prevention strategies but developers used different classification systems to indicate the levels of evidence across the studied guidelines. Of concern is the number of unresolved issues across the reviewed CPGs, which demonstrates substantial gaps in the research evidence base. Notably, very few recommendations were identified in relation to wound care strategies, which is indicative of the paucity of robust evidence.[35] This partly explains the undesirable practice variation in wound care.[10, 36] Some experts [37] have warned against the increase in the use of low grade recommendations for which the evidence is inconclusive or weak. *Choosing Wisely* campaigns attempt to address this through specialty-specific lists of recommendations of ‘things that clinicians and patients should question’.[38]

Overall, %mean scores were higher in 4/6 domains: *scope and purpose*, *rigour of development*, *clarity of presentation*, and *editorial independence*. Our results are similar to other CPG reviews covering different clinical topics.[31, 39, 40] These results may reflect ongoing improvements in CPG methodology, which have advanced over the past decade. As the methodology for developing guidelines becomes more established, rigorous and accepted internationally, it will become more readily adopted. Consequently, the criteria used to assess CPGs—based on the methodology—will also improve.

In relation to *stakeholder involvement*, most included CPGs described the representation of various health professional groups. The inclusion of experts from different professional disciplines acknowledges the importance of a multidisciplinary and collaborative approach that is needed to implement interventions in the prevention of SSI.[41] Still, only two CPGs included patients and their representatives (i.e., or parents/guardians as representatives of patients’ welfare) in guideline development. One of the pillars of evidence-based medicine is patient-centeredness, which is manifest in care that is respectful of and responsive to the expectations, preferences and experiences of patients.[32, 42, 43] Ultimately, patient values and preferences should, where possible, inform clinical decisions.[43] The increasing uptake of Patient and Public Involvement (PPI) groups encourages research development processes to include patients so that the research is ‘by’ them and not just ‘about’ them[44, 45]. As such, guideline developers should consider integrating healthcare consumers in future CPG updates to make them even more comprehensive and relevant.

*Applicability* is critical in the implementation of a guideline. In our review, this domain scored much lower than the other five domains. A recent systematic review [46] of 20 studies including 137 guidelines that used AGREE to assess CPGs from 2008–13 found that applicability scored lower than all other domains, and did not significantly improve over time. It is important that guidelines can be adapted to suit different clinical and financial contexts. Clearly, the local context profoundly influences applicability, and will therefore have a significant impact on adoption of the guideline.[42] For instance, in SSI prevention it would be meaningless to recommend a practice or an intervention (e.g., use of pre-warming device, hair removal), if the device is not available or the practice contravenes the cultural norms of a specific setting/group. Nonetheless, this does not negate the imperative for having guidelines based on best evidence. Rather, it emphasises the *extent to which* evidence obtained in a specific setting is generally valid or applicable to other contexts or situations. While the AGREE II tool is designed to evaluate the overall methodological quality of CPGs, the Appraisal of Guidelines Research and Evaluation-Recommendations Excellence (AGREE-REX) [47] has been recently developed to supplement this tool. The AGREE-REX specifically evaluates guideline recommendations relative to trustworthiness, suitability and feasibility of implementation in a particular context. The AGREE-REX tool is still under going further refinement [47], but goes some way to addressing issues related to the local context.

In the reviewed CPGs, there was limited consideration of resource implications. Conversely, with few health economic studies undertaken to evaluate strategies in SSI prevention,[48] guideline developers are challenged to include evidence on the economic benefit of interventions in this field. Economic evaluations have the potential to provide evidence for what works *best* and for what works *most efficiently* in real-world practice settings, to ultimately inform healthcare decision-making.[49] Clearly, information deficiencies drive waste, and can lead to the overuse of interventions and treatments that are of little, if any value or benefit to patients.[38, 50]

### Strengths and limitations

As with all systematic reviews, we acknowledge some limitations. The inclusion of CPGs covering all phases of surgical care and across all specialties meant that we necessarily excluded high quality CPGs [10] that were more focussed and specific, and perhaps more user-friendly to busy clinicians, such as Ubbink *et al*.[10] Although our search methods were exhaustive and robust, we may have possibly missed other CPGs and updates. However, our extensive search strategy covered all indexed and grey literature, and used multiple appraisers who undertook training and calibration to assess the quality of the CPGs. We used an appraisal tool with established validity and reliability [14] and all reviewers independently appraised CPGs. However, there may be different levels of understanding of the AGREE II tool among appraisers. To address this, we held regular meetings to ensure consistency in the appraisal process across the included CPGs. These discussions offered appraisers the opportunity to present information overlooked by others in the team, therefore clarifying and increasing understanding of the criteria upon which to evaluate the CPGs. Finally, our research team comprised of healthcare professionals from varied professional disciplines, research expertise and experience, thus adding a deeper dimension to guideline interpretation and appraisal.

### Implications for translation

Implementation of evidence-based information remains a challenge in many healthcare contexts and it is often difficult to assess application and performance of a CPG in clinical practice. It takes approximately 5 years for any given CPG to be adopted into routine clinical practice and even the broadly accepted guidelines are often not fully followed.[32, 42] Multiple factors influence guideline use including patient, provider, institutional context and systems issues; yet implementation is meant to overcome these barriers.[32, 46] Implementation tools that increase guideline accessibility using a variety of user-friendly formats.[31] For instance, presenting information found in the CPGs as recommendations with evidence summaries, repositories for tools for implementation, and implementation plans and toolkits make guidelines more accessible. Developers should also consider the feasibility and acceptability of implementing SSI prevention interventions across patient groups and different clinical settings, including those in developing countries.

Good information is essential for *choosing wisely* clinical interventions and treatments in SSI prevention, thus avoiding wasteful healthcare. Most of the reviewed CPGs lacked information about cost-effectiveness and risk-benefit analyses of the strategies used in SSI prevention. Many of the SSI prevention interventions used across the pre-, intra- and post-operative periods have never been subjected to rigorous economic evaluation,[48] but nevertheless continue to be used in clinical practice. Thus, it is important to conduct rigorous parallel economic evaluations alongside trials of clinical effectiveness,[49] as this will provide greater guidance to healthcare decision and policy makers. In terms of applicability and implementation, the inclusion of cost analyses studies in CPGs will assist clinicians in selecting the best available evidence-based options in healthcare organisations with limited resources.[49, 51]

## Conclusions

Successful uptake of CPGs depends on clinicians and decision makers trusting the quality and credibility of the content, and on the information presented in an accessible and practical way. It is critical that SSI prevention practices reflect the best available evidence and that the evidence is as current as possible in the face of uncertainty and existing gaps in the current evidence base. Further, it is essential to include healthcare consumers such as patient representatives to ensure patients’ needs and preferences are considered during guideline development. Finally, developers need to consider the inherent challenges associated with implementation and sustainability as these have important implications for uptake and sustainability.

## Supporting information

S1 Checklist PRISMA 2009 checklist. (DOC)

S1 Table MEDLINE (EBSCO) search strategy and guideline repository searches. (DOCX)

S2 Table AGREE II domain definitions. (DOCX)

S3 Table Summary of sources where CPGs were obtained. (DOCX)

S4 Table Evidence level systems used across CPGs. (DOCX)

S5 Table Recommendations across all CPGs that informed Table 3. (DOCX)
