# Supplementary material for: Quality appraisal of clinical guidelines for surgical site infection prevention: A systematic review
Source: PLoS One. 2018 Sep 13;13(9):e0203354. doi: 10.1371/journal.pone.0203354 (PMC6136720; doi:10.1371/journal.pone.0203354)
Supplement: S1 Table — (DOCX) [file pone.0203354.s002.docx]

**S1 Table. MEDLINE (EBSCO) search strategy and guideline repository searches**

**MEDLINE (final search run day: 02/03/2018)**

| ***n*** | **Descriptor** | **Result *n*** |
| --- | --- | --- |
| 1 | (MH “Practice Guidelines as Topic”) OR (MH “Practice Guideline”) | 101,385 |
| 2 | (MH “Consensus”) | 8,257 |
| 3 | (MH “Practice Patterns, Physicians’”) OR (MH “Practice Patterns, Nurses’”) | 52,629 |
| 4 | TX practice guideline* | 138,559 |
| 5 | TX clinical guideline* | 34,624 |
| 6 | TX consensus statement* | 6,039 |
| 7 | TX consensus development* | 15,209 |
| 8 | TX practice pattern* | 60,566 |
| 9 | TX practice management guideline* | 2,163 |
| 10 | TX best practice* | 29,631 |
| 11 | (S1 OR S2 OR S3 OR S4 OR S5 OR S6 OR S7 OR S8 OR S9 OR S10) | 244,810 |
| 12 | (MH “Surgical Wound”) OR (MH “Surgical Wound Infection/SU”) | 2,380 |
| 13 | TX ssi | 5,205 |
| 14 | TX operat* NEAR/3 wound* OR site* | 1,357,696 |
| 15 | TX surg* NEAR/3 infect* OR site* | 1,357,696 |
| 16 | (S12 OR S13 OR S14 OR S15) | 1,361,803 |
| 17 | TX manag* | 1,469,324 |
| 18 | TX prevent* | 2,255,853 |
| 19 | (S17 OR S18) | 3,495,769 |
| 20 | (MH “Infection/SU”) | 349 |
| 21 | TX infect* | 2,296,871 |
| 22 | (S20 OR S21) | 2,296,871 |
| 23 | (S11 AND S16 AND S19 AND S22)  [Limits: Date of Publication: 1990-2018; English Language; Human] | 944 |

TX denotes full-text search

**Guidelines Repository searches**

| **Repository name** | **Search term(s) used** | **Total search results** | **Eligible CPGs** |
| --- | --- | --- | --- |
| The National Guideline Clearinghouse | “surgical site infection guidelines”  (limited to Clinical specialty – ‘Surgery’) | 90 | 1 – Surgical site infection: prevention and treatment of surgical site infection. National Guidelines Clearinghouse summary of NICE, 2008 guidelines.  2 – Global guidelines for the prevention of surgical site infection. National Guidelines Clearinghouse summary of WHO, 2016 guidelines.  Total = 2 |
| National Institute for Health and Care Excellence (NICE) | “surgical site infection prevention guidelines”  (limited to Evidence type – ‘Guidance’; Guidance category – ‘Clinical guidelines’) | 15 | 1 – Surgical site infections: prevention and treatment: guidance (CG74), 2008 (NICE, UK)  Total = 1 |
| National Health and Medical Research Council (NHMRC) – Australian Clinical Practice Guidelines | “surgical site infection” | 0 | 0 |
| CPG Infobase: Clinical Practice Guidelines (Canadian Medical Association) | “surgical site infection” | 1 | 0 |
| Scottish Intercollegiate Guideline Network (SIGN) | Nil – search from a list of guidelines provided | 56 | 0 |
| New Zealand Guidelines Group | “surgical site infection” | 3 | 0 |
| Clinical Key Elsevier | “surgical site infection”  (limited to “guidelines”) | 114 | 1 – Surgical site infection: prevention and treatment (CG74). National Guidelines Clearinghouse summary of NICE, 2008 guidelines.  2- Strategies to Prevent Surgical Site Infections in Acute Care Hospitals: 2014 Update (Anderson et al, 2014)  Total = 2 |
| BMJ Best Practice | “surgical infection”  (limited to “guidelines”) | 0 | 0 |
| **TOTALS** | **-** | **289** | **3** |
