# Supplementary material for: Quality appraisal of clinical guidelines for surgical site infection prevention: A systematic review
Source: PLoS One. 2018 Sep 13;13(9):e0203354. doi: 10.1371/journal.pone.0203354 (PMC6136720; doi:10.1371/journal.pone.0203354)
Supplement: S2 Table — (DOCX) [file pone.0203354.s003.docx]

**S3 Table. AGREE II domain definitions**

**Overall assessment**: This is a rating of the overall quality of the guideline, based on the judgement of guideline appraisers, and dictates whether the appraiser would recommend the use of the guideline in practice.

**Domain 5 – Editorial Independence**: This domain is concerned with the formulation of recommendations not being unduly biased with competing interest, such as funding, personal gain or ghost writing (items 22-23).

**Domain 5 – Applicability**: This domain pertains to the likely barriers and facilitators to guideline implementation, strategies to improve and monitor guideline uptake, and the resource implications of applying the guideline (item 18-21).

**Domain 4 – Clarity of Presentation**: This domain focusses on the language, structure and the format of the guideline (items 15-17).

**Domain 3 – Rigour of Development**: This domain relates to the processes used to gather and synthesise evidence that underpins the guideline, the methods used to formulate recommendations, and the process for updating the guideline (items 7-14).

**Domain 2 – Stakeholder Involvement**: This domain focuses on the extent to which the guideline was developed by the appropriate stakeholders and consequently, how well the guideline represents the views of its’ intended users (items 4-6).

**Domain 1 – Scope and Purpose**: This domain is concerned with the overall aim(s) of the guideline, the specific health question(s) it attempts to address and the target population(s) that the guideline focusses on (items 1-3).

Brouwers MC, Kho ME, Browman GP, et al.; The AGREE Next Steps Consortium. AGREE II: Advancing guideline development, reporting and evaluation in healthcare. *CMAJ* 2010. doi:10.1503/cmaj.090449
