# Supplementary material for: Quality appraisal of clinical guidelines for surgical site infection prevention: A systematic review
Source: PLoS One. 2018 Sep 13;13(9):e0203354. doi: 10.1371/journal.pone.0203354 (PMC6136720; doi:10.1371/journal.pone.0203354)
Supplement: S3 Table — (DOCX) [file pone.0203354.s004.docx]

**S3 Table – Summary of sources where CPGs were obtained**

| **CPG Document** | **Where sourced** | **Link to document** |
| --- | --- | --- |
| CDC 1999 CPG | EMBASE | <https://stacks.cdc.gov/view/cdc/7160> |
| CDC 2017 CPG update | Hand-searching | <https://jamanetwork.com/journals/jamasurgery/fullarticle/2623725> |
| CDC 2017 CPG supplementary online content | Hand-searching | <https://jamanetwork.com/journals/jamasurgery/fullarticle/2623725> |
| NICE 2008 CPG | The National Guideline Clearinghouse; National Institute for Health and Care Excellence (NICE); Clinical Key Elsevier and MEDLINE | <https://www.nice.org.uk/guidance/cg74> |
| NICE 2014 CPG update | Hand-searching |  |
| WHO 2016 CPG | MEDLINE and The National Guideline Clearinghouse | <https://www.guideline.gov/summaries/summary/50706/global-guidelines-for-the-prevention-of-surgical-site-infection?q=WHO+surgical+site+infection> |
| Strategies to Prevent SSI 2008 CPG | EMBASE (had to be acquired through Griffith inter-library loan) | n/a (available upon request) |
| Strategies to Prevent SSI 2008 CPG executive summary | Acquired through hand-searching and Griffith inter-library loan | n/a (available upon request) |
| Strategies to Prevent SSI 2008 CPG introduction | Acquired through hand-searching and Griffith inter-library loan | n/a (available upon request) |
| Strategies to Prevent SSI 2014 CPG update | EMBASE and Clinical Key Elsevier | <https://www-cambridge-org.libraryproxy.griffith.edu.au/core/services/aop-cambridge-core/content/view/EE4D1EC09206F231C69CB0E1A3F4EAC9/S0899823X00193869a.pdf/strategies_to_prevent_surgical_site_infections_in_acute_care_hospitals_2014_update.pdf> |
| Strategies to Prevent SSI 2014 CPG compendium | Acquired through hand-searching and Griffith inter-library loan | n/a (available upon request) |
| Strategies to Prevent SSI 2014 CPG introduction | Acquired through hand-searching and Griffith inter-library loan | n/a (available upon request) |
| ACS SIS 2017 CPG | Acquired through hand-searching and Griffith inter-library loan | <http://www.sciencedirect.com/science/article/pii/S1072751516315630?via%3Dihub> |
| ACS SIS 2017 CPG executive summary | EMBASE | <http://online.liebertpub.com.libraryproxy.griffith.edu.au/doi/pdfplus/10.1089/sur.2016.214> |
| University of Toronto 2017 CPG | Acquired through hand-searching | <http://bestpracticeinsurgery.ca/wp-content/uploads/2017/11/SSI-BPS-CPG-Nov20.pdf> |
