# Supplementary material for: Quality appraisal of clinical guidelines for surgical site infection prevention: A systematic review
Source: PLoS One. 2018 Sep 13;13(9):e0203354. doi: 10.1371/journal.pone.0203354 (PMC6136720; doi:10.1371/journal.pone.0203354)
Supplement: S4 Table — (DOCX) [file pone.0203354.s005.docx]

**S4 Table – Evidence level systems used across CPGs**

| **Evidence Levels** | **CPG Working Group** |
| --- | --- |
| **American College of Surgeons/Surgical Infection Society (2016)** | |
| **WG** | Recommendations based on expert opinion/consensus by the working group. |
| **Strategies to Prevent SSI (2008)** | |
| **A** | Good evidence to support a recommendation for use. |
| **B** | Moderate evidence to support a recommendation for use. |
| **C** | Poor evidence to support a recommendation. |
| **I** | Evidence from ≥1 properly randomised, controlled trial. |
| **II** | Evidence from ≥1 well-designed clinical trial, without randomisation; from cohort or case-control analytic studies (preferably from >1 center): from multiple time series; or from dramatic results from uncontrolled experiments. |
| **III** | Evidence from opinions of respected authorities, based on clinical experience, descriptive studies, or reports of expert committees. |
| **Strategies to Prevent SSI (2014)** | |
| **I (High)** | Highly confident that the true effect lies close to that of the estimated size and direction of the effect. Evidence is rated as high quality when there is a wide range of studies with no major limitations, there is little variation between studies, and the summary estimate has a narrow confidence interval. |
| **II (Moderate)** | The true effect is likely to be close to the estimated size and direction of the effect, but there is a possibility that it is substantially different. Evidence is rated as moderate quality when there are only a few studies and some have limitations but not major flaws, there is some variation between studies, or the confidence interval of the summary estimate is wide. |
| **III (Low)** | The true effect may be substantially different from the estimated size and direction of the effect. Evidence is rated as low quality when supporting studies have major flaws, there is important variation between studies, the confidence interval of the summary estimate is very wide, or there are no rigorous studies, only expert consensus. |
| **CDC (1999)** | |
| **1A** | Strongly recommended for implementation and supported by well-designed experimental, clinical, or epidemiological studies. |
| **1B** | Strongly recommended for implementation and supported by some experimental, clinical, or epidemiological studies and strong theoretical rationale. |
| **2** | Suggested for implementation and supported by suggestive clinical or epidemiological studies or theoretical rationale. |
| **No recommendation/ unresolved issue** | Practices for which insufficient evidence or no consensus regarding efficacy exists. |
| **CDC (2017)** | |
| **1A** | A strong recommendation supported by high to moderate–quality evidence suggesting net clinical benefits or harms |
| **1B** | A strong recommendation supported by low-quality evidence suggesting net clinical benefits or harms or an accepted practice (e.g., aseptic technique) supported by low to very low– quality evidence. |
| **1C** | A strong recommendation required by state or federal regulation. |
| **2** | A weak recommendation supported by any quality evidence suggesting a trade-off between clinical benefits and harms. |
| **No recommendation/**  **unresolved issue** | An issue for which there is low to very low–quality evidence with uncertain trade-offs between the benefits and harms or no published evidence on outcomes deemed critical to weighing the risks and benefits of a given intervention. |
| **NICE (2008 and 2014)** | |
| **1++** | High-quality meta-analyses, systematic reviews of RCTs, or RCTs with a very low risk of bias |
| **1+** | Well-conducted meta-analyses, systematic reviews of RCTs, or RCTs with a low risk of bias |
| **1-** | Meta-analyses, systematic reviews of RCTs, or RCTs with a low risk of bias |
| **2++** | Well-conducted case-control or cohort studies with a low risk of confounding, bias or chance and a high probability that the relationship is causal |
| **2+** | Well-conducted case-control or cohort studies with a low risk of confounding, bias or chance and a moderate probability that the relationship is causal |
| **2-** | Case-control or cohort studies with a high risk of confounding, bias or chance and a significant risk that the relationship is not causal |
| **3** | Non-analytical studies (for example, case reports, case series) |
| **4** | Expert opinion, formal consensus |
| **WHO (2016) and University of Toronto (2017)** | |
| **High quality of evidence** | We are very confident that the true effect lies close to that of the estimate of the effect. |
| **Moderate quality of evidence** | We are moderately confident in the effect estimate: the true effect is likely to be close to the estimate of the effect, but there is a possibility that it is substantially different. |
| **Low quality of evidence** | Our confidence in the effect estimate is limited: the true effect may be substantially different from the estimate of the effect. |
| **Very low quality of evidence** | We have very little confidence in the effect estimate: the true effect is likely to be substantially different from the estimate of the effect. |
