# Supplementary material for: Quality appraisal of clinical guidelines for surgical site infection prevention: A systematic review
Source: PLoS One. 2018 Sep 13;13(9):e0203354. doi: 10.1371/journal.pone.0203354 (PMC6136720; doi:10.1371/journal.pone.0203354)
Supplement: S5 Table — (DOCX) [file pone.0203354.s006.docx]

**S5 Table – Specific recommendations across all CPGs that informed Table 3**

| **Recommendations identified in Table 3** | **Corresponding recommendation from each of the included CPGs** |
| --- | --- |
| **PRE-OPERATIVE PHASE** | |
| 1. Showering/bathing | *NICE (2008):*  • Advise patients to shower or have a bath (or help patients to shower, bath or bed bath) using soap, either the day before, or on the day of, surgery (1+).  *NICE (2014 update):*  • No further recommendation, current recommendation stands  *WHO (2016):*  • It is good clinical practice for patients to bathe or shower prior to surgery (Moderate).  • The panel suggests that either plain soap or an antimicrobial soap may be used for this purpose (Moderate).  • The panel decided not to formulate a recommendation on the use of CHG impregnated cloths for the purpose of reducing SSI due to the very low quality of evidence (Moderate).  *CDC (1999):*  • Require patients to shower or bathe with an antiseptic agent on at least the night before the operative day (Category IB).  • Thoroughly wash and clean at and around the incision site to remove gross contamination before performing antiseptic skin preparation (Category IB).  *CDC (2017 update):*  • Advise patients to shower or bathe (full body) with soap (antimicrobial or non-antimicrobial) or an antiseptic agent on at least the night before the operative day (Category IB–strong recommendation; accepted practice).  • Randomized controlled trial evidence suggested uncertain trade-offs between the benefits and harms regarding the optimal timing of the preoperative shower or bath, the total number of soap or antiseptic agent applications, or the use of chlorhexidine gluconate washcloths for the prevention of SSI (No recommendation/ unresolved issue).  *ASC SIS (2016):*  • Routine preoperative bathing with chlorhexidine (when not part of a decolonization protocol or preoperative bundle) decreases skin surface pathogen concentrations, but has not been shown to reduce SSI (WG).  *University of Toronto (2017):*   - Patients should bathe or shower the entire body and head prior to surgery using plain or antimicrobial soap (WG). |
| 2. Hair removal | *Strategies to Prevent SSI (2008):*  • Do not remove hair unless hair will interfere will interfere with the operation; if hair removal is necessary, remove by clipping and do not use razors (A-I).  *Strategies to Prevent SSI (2014 update):*  • Do not remove unless hair will interfere with the operation. If hair removal is necessary; remove outside the OR by clipping. Do not use razors (II).  *NICE (2008):*  • Do not use hair removal routinely to reduce the risk of surgical site infection (1+).  • If hair has to be removed, use electric clippers with a single-use head on the day of surgery. Do not use razors for hair removal, because they increase the risk of surgical site infection (1+)*.*  *NICE (2014 update):*  • No further recommendation, current recommendation stands  *WHO (2016):*  • The panel recommends that in patients undergoing any surgical procedure, hair should either not be removed or, if absolutely necessary, should be removed only with a clipper. Shaving is strongly discouraged at all times, whether preoperatively or in the OR (Moderate).  *CDC (1999):*  • Do not remove hair preoperatively unless the hair at or around the incision site will interfere with the operation (Category IA).  - If hair is removed, remove immediately before the operation, preferably with electric clippers (Category IA).  *ASC SIS (2016):*  • Hair removal should be avoided unless hair interferes with surgery.  • If hair removal is necessary, clippers should be used instead of a razor.  *University of Toronto (2017):*   - Hair removal should not be performed for the purposes of SSI prevention. If hair removal is necessary, clippers should be used (Level of evidence: Moderate). |
| 3. Antibiotic prophylaxis | *Strategies to Prevent SSI Strategies to Prevent SSI (2008):*  • Increase dosing of prophylactic antimicrobial agents for morbidly obese patients (A-II).  • Administer only when indicated (A-I).  • Select appropriate agents on the basis of surgical procedure, most common pathogens causing SSI for a specific procedure and published recommendations (A-I).  • Do not routinely use vancomycin for antimicrobial prophylaxis (B-II).  *Strategies to Prevent SSI (2014 update):*  • Increase dosing of prophylactic antimicrobial agents for morbidly obese patients (I).  • Administer only when indicated (I).  • Select appropriate agents on the basis of surgical procedure, most common pathogens causing SSIs for a specific procedure, and published recommendations (I).  • Do not routinely use vancomycin for antimicrobial prophylaxis (II).  *NICE (2008):*  • Give antibiotic prophylaxis to patients before:  • clean surgery involving the placement of a prosthesis or implant  • clean-contaminated surgery  • contaminated surgery  • Do not use antibiotic prophylaxis routinely for clean non-prosthetic uncomplicated surgery.  • Use the local antibiotic formulary and always consider potential adverse effects when choosing specific antibiotics for prophylaxis.  • Consider giving a single dose of antibiotic prophylaxis intravenously on starting anaesthesia. However, give prophylaxis earlier for operations in which a tourniquet is used.  • Give antibiotic treatment (in addition to prophylaxis) to patients having surgery on a dirty or infected wound.  • Inform patients before the operation, whenever possible, if they will need antibiotic prophylaxis, and afterwards if they have been given antibiotics during their operation.  (Evidence ranging from: 1+ — 1-)  *NICE (2014 update):*  • No further recommendation, current recommendation stands  *WHO (2016):*  • The panel recommends that SAP should be administered prior to the surgical incision when indicated (depending on the type of operation) (Low).  • The panel recommends the administration of SAP within 120 minutes before incision, while considering the half-life of the antibiotic (Moderate).  *CDC (1999):*  • Administer a prophylactic antimicrobial agent only when indicated, and select it based on its efficacy against the most common pathogens causing SSI for a specific operation and published recommendations (Category IA).  • Administer by the intravenous route the initial dose of prophylactic antimicrobial agent, timed such that a bactericidal concentration of the drug is established in serum and tissues when the incision is made. Maintain therapeutic levels of the agent in serum and tissues throughout the operation and until, at most, a few hours after the incision is closed in the operating room (Category IA).  • Do not routinely use vancomycin for antimicrobial prophylaxis (Category IB).  *CDC (2017 update):*  • Administer preoperative antimicrobial agents only when indicated based on published clinical practice guidelines and timed such that a bactericidal concentration of the agents is established in the serum and tissues when the incision is made (Category IB–strong recommendation; accepted practice).  • No further refinement of timing can be made for preoperative antimicrobial agents based on clinical outcomes (No recommendation/unresolved issue).  • Administer the appropriate parenteral prophylactic antimicrobial agents before skin incision in all cesarean section procedures (Category IA–strong recommendation; high-quality evidence).  • The literature search did not identify randomized controlled trials that evaluated the benefits and harms of weight-adjusted parenteral antimicrobial prophylaxis dosing and its effect on the risk of SSI. Other organizations have made recommendations based on observational and pharmacokinetic data, and a summary of these recommendations can be found in the Other Guidelines section of the narrative summary for this question (No recommendation/unresolved issue).  • The search did not identify sufficient randomized controlled trial evidence to evaluate the benefits and harms of intraoperative re- dosing of parenteral prophylactic antimicrobial agents for the prevention of SSI. Other organizations have made recommendations based on observational and pharmacokinetic data, and a summary of these recommendations can be found in the Other Guidelines section of the narrative summary for this question (No recommendation/unresolved issue).  • In clean and clean-contaminated procedures, do not administer additional prophylactic antimicrobial agent doses after the surgical incision is closed in the operating room, even in the presence of a drain (Category IA–strong recommendation; high-quality evidence).  *ASC SIS (2016):*  • Administer prophylactic antibiotics only when indicated (WG).  • Choice of prophylactic antibiotic should be dictated by the procedure and pathogens most likely to cause SSI (WG).  • Prophylactic antibiotic should be administered within 1 hour before incision or within 2 hours for vancomycin or fluoroquinolones (WG).  • Prophylactic antibiotic dosing should be weight-adjusted (WG).  • Re-dose antibiotics to maintain adequate tissue levels based on agent half-life or for every 1,500 mL blood loss (WG).  • There is no evidence that prophylactic antibiotic administration after incision closure decreases SSI risk; prophylactic antibiotics should be discontinued at time of incision closure (exceptions include implant- based breast reconstruction, joint arthroplasty, and cardiac procedures where optimal duration of antibiotic therapy remains unknown) (WG).  *University of Toronto (2017):*   - All patients having surgery should receive appropriate prophylactic antibiotics except for some clean surgical procedures (Level of evidence: High). - Antibiotics should be dosed to optimize tissues concentrations (Level of evidence: Moderate) - Patients receiving therapeutic antibiotics preoperatively are at increased risk for surgical site infections. The optimal method of prophylaxis is unknown, but unless the therapeutic antibiotic provides coverage for SSI prophylaxis, prophylactic antibiotics should be administered. As well, these antibiotics should be timed to ensure maximal tissue concentration at incision (Level of evidence: Very low) |
| 4. Nasal decontamination | *NICE (2008):*  • Do not use nasal decontamination with topical antimicrobial agents aimed at eliminating *Staphylococcus aureus* routinely to reduce the risk of surgical site infection (1+).  *WHO (2016):*  • The panel recommends that patients undergoing cardiothoracic and orthopaedic surgery with known nasal carriage of *S. aureus* should receive perioperative intranasal applications of mupirocin 2% ointment with or without a combination of CHG body wash (Moderate).  • The panel suggests considering to treat also patients with known nasal carriage of *S. aureus* undergoing other types of surgery with perioperative intranasal applications of mupirocin 2% ointment with or without a combination of CHG body wash (Moderate).  *CDC (1999):*  • No recommendation to preoperatively apply mupirocin to nares to prevent SSI (Unresolved issue). |
| 5. Mechanical bowel preparation | *NICE (2008):*  • Do not use mechanical bowel preparation routinely to reduce the risk of surgical site infection (1+).  *WHO (2016):*  • The panel suggests that preoperative oral antibiotics combined with mechanical bowel preparation should be used to reduce the risk of SSI in adult patients undergoing elective colorectal surgery (Moderate).  • The panel recommends that mechanical bowel preparation alone (without administration of oral antibiotics) should not be used for the purpose of reducing SSI in adult patients undergoing elective colorectal surgery (Moderate).  *CDC (1999):*  • Before elective colorectal operations in addition to d2 above, mechanically prepare the colon by use of enemas and cathartic agents. Administer non- absorbable oral antimicrobial agents in divided doses on the day before the operation (Category IA).  *ASC SIS (2016):*  • Combination mechanical and antibiotic (po) preparation is recommended for all elective colectomies (WG). |
| 6. Surgical site antimicrobial skin preparation | *Strategies to Prevent SSI (2008):*  • Wash and clean skin around incision site; use an appropriate antiseptic agent (A-II).  *Strategies to Prevent SSI (2014 update):*  • Wash and clean skin around incision site. Use a dual agent skin preparation containing alcohol, unless contraindications exist (I).  *WHO (2016):*  • The panel recommends alcohol-based antiseptic solutions based on CHG for surgical site skin preparation in patients undergoing surgical procedures (Low-moderate).  • The panel suggests that antimicrobial sealants should not be used after surgical site skin preparation for the purpose of reducing SSI (Very low).  *CDC (1999):*  • Use an appropriate antiseptic agent for skin preparation (Category IB).  • Apply preoperative antiseptic skin preparation in concentric circles moving toward the periphery. The prepared area must be large enough to extend the incision or create new incisions or drain sites, if necessary (Category II).  *ASC SIS (2016):*  • Alcohol-containing preparation should be used unless contraindication exists (eg fire hazard, surfaces involving mucosa, cornea, or ear).  • No clear superior agent (chlorhexidine vs iodine) when combined with alcohol.  • If alcohol cannot be included in the preparation, chlorhexidine should be used instead of iodine unless contraindications exist.  *University of Toronto (2017):*   - Patients should be prepped with alcohol-based chlorhexidine gluconate (2% chlorhexidine gluconate and 70% isopropyl alcohol) with the following exceptions: (i) povidone iodine should continue to be used for ophthalmic procedures and those involving the inner ear or mucous membranes; (ii) procedures where there is not time for alcohol solutions to dry (e.g. in trauma), an aqueous-based antiseptic solution should be used and allowed to dry; and (iii) infants less than 2 months old (Level of evidence: Moderate) - Alcohol-based antiseptics are flammable in operative procedures involving electrosurgery (i.e. electrocautery) so pooling on drapes and the patient should be avoided. The antiseptic solution should also be allowed time to dry completely (~ 3 minutes, longer in areas with excess hair) to limit fire hazard (Level of evidence: Low) |
| 7. Patient theatre attire | *NICE (2008):*  • Give patients specific theatre wear that is appropriate for the procedure and clinical setting and that provides easy access to the operative site and areas for placing devices, such as intravenous cannulas. Consider also the patient’s comfort and dignity (4). |
| **PRE-OPERATIVE AND/OR INTRAOPERATIVE PHASES** | |
| 1. *Patient Homeostasis:* |  |
| - Glycaemic control | *Strategies to Prevent SSI (2008):*  • Control serum BGLs; reduce HbA1c levels to <7% before surgery if possible (A-II).  *Strategies to Prevent SSI (2014 update):*  • Control serum blood glucose levels for all surgical patients, including patients without diabetes. For patients with diabetes mellitus, reduce glycosylated hemoglobin Ale levels to less than 7% before surgery, if possible (I).  *NICE (2008):*  • Do not give insulin routinely to patients who do not have diabetes to optimise blood glucose postoperatively as a means of reducing the risk of surgical site infection (1-).  *WHO (2016):*  • The panel suggests the use of protocols for intensive perioperative blood glucose control for both diabetic and non-diabetic adult patients undergoing surgical procedures to reduce the risk of SSI (Low).  *CDC (1999):*  • Adequately control serum blood glucose levels in all diabetic patients and particularly avoid hyperglycaemia perioperatively (Category IB).  *CDC (2017 update):*  • Implement perioperative glycemic control and use blood glucose target levels less than 200 mg/dL in patients with and without diabetes (Category IA–strong recommendation; high to moderate–quality evidence).  • The search did not identify randomized controlled trials that evaluated lower (<200 mg/dL) or narrower blood glucose target levels than recommended in this guideline nor the optimal timing, duration, or delivery method of perioperative glycemic control for the prevention of SSI. Other organizations have made recommendations based on observational evidence, and a summary of these recommendations can be found in the Other Guidelines section of the narrative summary for this question (No recommendation/unresolved issue).  • The search did not identify randomized controlled trials that evaluated the optimal hemoglobin A1C target levels for the prevention of SSI in patients with and without diabetes (No recommendation/unresolved issue).  *ASC SIS (2016):*  • Optimal blood glucose control should be encouraged for all diabetic patients; however, there is no evidence that improved HgbA1C decreases SSI risk (WG).  • Hyperglycemia in the immediate preoperative period is associated with an increased risk of SSI (WG).  • Target perioperative blood glucose should be between 110 to 150 mg/dL in all patients, regardless of diabetic status, except in cardiac surgery patients where the target perioperative blood glucose is <180 mg/dL (WG).  • Target blood glucose rates <110 mg/dL have been tied to adverse outcomes and increased episodes of hypoglycemia and do not decrease SSI risk (WG). |
| - Enhanced nutritional support | *Strategies to Prevent SSI (2008):*  • Do not routinely delay surgery to provide parenteral nutrition (A-I).  *Strategies to Prevent SSI (2014 update):*  • Do not routinely delay surgery to provide parenteral Nutrition (I).  *WHO (2016):*  • The panel suggests considering the administration of oral or enteral multiple nutrient-enhanced nutritional formulas for the purpose of preventing SSI in underweight patients who undergo major surgical operations (Very low).  *CDC (1999):*  • No recommendation to enhance nutritional sup- port for surgical patients solely to prevent SSI (Unresolved issue). |
| - Pre-warming | *NICE (2008):*  • Maintain patient temperature in line with ‘Inadvertent perioperative hypothermia’ (NICE clinical guideline 65).  *ASC SIS (2016):*  • Maintain intraoperative normothermia to reduce SSI risk. Preoperative warming is recommended for all cases, and intraoperative warming methods should be employed for all but short, clean cases (WG).  *University of Toronto (2017):*   - If the patient is at high risk of hypothermia or if his/her temperature is less than 36°C preoperatively, forced-air warmers should be started prior to induction to ensure a body temperature greater than 36°C prior to surgery (Level of evidence: Moderate) - The use of forced-air warming systems (Level of evidence: Moderate) and warmed IV and irrigation fluids (Level of evidence: Very low) should be used intraoperatively to maintain body temperature greater than 36°C for patients during the surgical procedure - Warming systems should not be used in patients undergoing surgery where intraoperative hypothermia is intended (i.e. off-pump surgery) (WG). |
| - Oxygenation | *Strategies to Prevent SSI (2014 update):*  • Optimize tissue oxygenation by administering supplemental oxygen during and immediately following surgical procedures involving mechanical ventilation (I).  *NICE (2008):*  • Maintain optimal oxygenation during surgery. In particular, give patients sufficient oxygen during major surgery and in the recovery period to ensure that a haemoglobin saturation of more than 95% is maintained (1+, 1-).  *NICE (2014 update):*  • No further recommendation  *WHO (2016):*  • The panel recommends that adult patients undergoing general anaesthesia with endotracheal intubation for surgical procedures should receive an 80% fraction of inspired oxygen intraoperatively and, if feasible, in the immediate postoperative period for 2-6 hours to reduce the risk of SSI (Moderate).  *CDC (1999):*  • No recommendation to provide measures that enhance wound space oxygenation to prevent SSI (Unresolved issue).  *CDC (2017 update):*  • Randomized controlled trial evidence suggested uncertain trade- offs between the benefits and harms regarding the administration of increased fraction of inspired oxygen (FIO2) via endotracheal intubation during only the intraoperative period in patients with normal pulmonary function undergoing general anesthesia for the prevention of SSI (No recommendation/unresolved issue).  • For patients with normal pulmonary function undergoing general anesthesia with endotracheal intubation, administer increased FIO2 during surgery and after extubation in the immediate postoperative period. To optimize tissue oxygen delivery, maintain perioperative normothermia and adequate volume replacement (Category IA–strong recommendation; moderate-quality evidence).  • Randomized controlled trial evidence suggested uncertain trade- offs between the benefits and harms regarding the administration of increased FIO2 via face mask during the perioperative period in patients with normal pulmonary function undergoing general anesthesia without endotracheal intubation or neuraxial anesthesia (ie, spinal, epidural, or local nerve blocks) for the prevention of SSI (No recommendation/unresolved issue).  • Randomized controlled trial evidence suggested uncertain trade-offs between the benefits and harms regarding the administration of increased FIO2 via face mask or nasal cannula during only the postoperative period in patients with normal pulmonary function for the prevention of SSI (No recommendation/ unresolved issue).  • The search did not identify randomized controlled trials that evaluated the optimal target level, duration, and delivery method of FIO2 for the prevention of SSI. Other organizations have made recommendations based on observational studies, and a summary of these recommendations can be found in the Other Guidelines section of the narrative summary for this question (No recommendation/unresolved issue). |
| - Discontinuation of immune-suppressants | *Strategies to Prevent SSI (2008):*  • No formal recommendation; in general, avoid immunosuppressive medications in perioperative period if possible (C-II).  *Strategies to Prevent SSI (2014 update):*  • Avoid immune-suppressive medications in perioperative period, if possible (III).  *WHO (2016):*  • The panel suggests not to discontinue immunosuppressive medication prior to surgery for the purpose of preventing SSI (Very low).  *CDC (1999):*  • No recommendation to taper or discontinue systemic steroid use (when medically permissible) before elective operation (Unresolved issue).  *CDC (2017 update – Ortho):*   - Available evidence suggested uncertain trade-offs between the benefits and harms of systemic corticosteroid or other immunosuppressive therapies on the risk of SSI in prosthetic joint arthroplasty. Other organizations have made recommendations based on the existing evidence, and a summary of these recommendations can be found in the Other Guidelines section of the narrative summary for this question (eAppendix 1 of the Supplement). (No recommendation/unresolved issue.) - For prosthetic joint arthroplasty patients receiving systemic corticosteroid or other immunosuppressive therapy, recommendation 1E applies: in clean and clean-contaminated procedures, do not administer additional antimicrobial prophylaxis doses after the surgical incision is closed in the operating room, even in the presence of a drain. (Category IA–strong recommendation; high quality evidence.) |
| **INTRAOPERATIVE PHASE** | |
| 1. Optimal timing for antibiotics | *Strategies to Prevent SSI (2008):*  • Administer within 1-hour before incision to maximise tissue concentration (A-I).  *Strategies to Prevent SSI (2014 update):*  • Administer within 1 hour of incision to maximize tissue concentration (I).  *NICE (2008):*  • Before giving antibiotic prophylaxis, consider the timing and pharmacokinetics (for example, the serum half-life) and necessary infusion time of the antibiotic. Give a repeat dose of antibiotic prophylaxis when the operation is longer than the half-life of the antibiotic given (2+).  *CDC (1999):*  • Administer by the intravenous route the initial dose of prophylactic antimicrobial agent, timed such that a bactericidal concentration of the drug is established in serum and tissues when the incision is made. Maintain therapeutic levels of the agent in serum and tissues throughout the operation and until, at most, a few hours after the incision is closed in the operating room (Category IA).  *CDC (2017 update):*  • Administer preoperative antimicrobial agents only when indicated based on published clinical practice guidelines and timed such that a bactericidal concentration of the agents is established in the serum and tissues when the incision is made (Category IB–strong recommendation; accepted practice).  • No further refinement of timing can be made for preoperative antimicrobial agents based on clinical outcomes (No recommendation/unresolved issue).  *University of Toronto (2017):*   - Antibiotics should be administered within 60 minutes before surgical incision/tourniquet inflation. Vancomycin and fluoroquinolones require a longer infusion time and should be initiated to ensure completion within 60 minutes of incision (Level of evidence: Low-Moderate). - Antibiotics should be re-dosed if the duration of the procedure exceeds two half-lives of the antibiotic or there is excessive blood loss (>1.5L in adults) for all antibiotics except vancomycin (Level of evidence: Very low) |
| 2. Surgical attire, incisor drapes and gowns | *Strategies to Prevent SSI (2014 update):*  • All members of the operative team should double glove and change gloves when perforation is noted (III).  • Do not routinely use antiseptic drapes as a strategy to prevent SSIs (I).  *NICE (2008):*  • All staff should wear specific non-sterile theatre wear in all areas where operations are undertaken (1+-4).  • The operating team should remove hand jewellery before operations (1+).  • The operating team should remove artificial nails and nail polish before operations (4).  • Do not use non-iodophor-impregnated incise drapes routinely for surgery as they may increase the risk of surgical site infection (1+).  • If an incise drape is required, use an iodophor-impregnated drape unless the patient has an iodine allergy (1+).  • The operating team should wear sterile gowns in the operating theatre during the operation (4).  • Consider wearing two pairs of sterile gloves when there is a high risk of glove perforation and the consequences of contamination may be serious (4).  *NICE (2014 update):*  • No further recommendation, current recommendation stands  *WHO (2016):*  • The panel suggests that either sterile, disposable non-woven or sterile, reusable woven drapes and  gowns can be used during surgical operations for the purpose of preventing SSI (Very low – Moderate).  • The panel suggests not to use plastic adhesive incise drapes with or without antimicrobial properties or the purpose of preventing SSI (Very low – Low).  *CDC (1999):*  • Wear a surgical mask that fully covers the mouth and nose when entering the operating room if an operation is about to begin or already under way, or if sterile instruments are exposed. Wear the mask throughout the operation (Category IB).  • Wear a cap or hood to fully cover hair on the head and face when entering the operating room (Category IB).  • Do not wear shoe covers for the prevention of SSI (Category IB).  • Wear sterile gloves if a scrubbed surgical team member. Put on gloves after donning a sterile gown (Category IB).  • Use surgical gowns and drapes that are effective barriers when wet (i.e., materials that resist liquid penetration) (Category IB).  • Change scrub suits that are visibly soiled, contaminated, and/or penetrated by blood or other potentially infectious materials (Category IB).  • No recommendations on how or where to launder scrub suits, on restricting use of scrub suits to the operating suite, or for covering scrub suits when out of the operating suite (Unresolved issue).  *CDC (2017 update):*  • The use of plastic adhesive drapes with or without antimicrobial properties is not necessary for the prevention of SSI (Category II–weak recommendation; high to moderate–quality evidence suggesting a trade-off between clinical benefits and harms).   - (Ortho) Available evidence suggested uncertain trade-offs between the benefits and harms of orthopedic space suits or the health care personnel who should wear them for the prevention of SSI in prosthetic joint arthroplasty. (No recommendation/unresolved issue.)   *ASC SIS (2016):*  • There is limited evidence to support recommendations on surgical attire. Joint Commission and Association of Perioperative Registered Nurses policies support facility scrub laundering and the use of disposable bouffant hats (WG).  • American College of Surgeons guidelines support the use of a skull cap if minimal hair is exposed, removing or covering all jewelry on the head and neck, and professional attire when outside the operating room (no scrubs or clean scrubs covered with a white coat) (WG).  • The use of double gloves is recommended. Changing gloves before closure in colorectal cases is recommended, however, rescrubbing before closure in colorectal cases is not recommended. |
| 3. Disposable versus reusable drapes and gowns | No recommendations made. |
| 4. Surgical scrub/hand antisepsis | *Strategies to Prevent SSI (2008):*  • Use appropriate antiseptic agent to perform 2-5 minutes preoperative surgical scrub or use an alcohol-based surgical hand antisepsis product (A-II).  *Strategies to Prevent SSI (2014 update):*  • Use appropriate antiseptic agent to perform preoperative surgical scrub. For most products, scrub the hands and forearms for 2-5 minutes (II).  • Adhere to standard principles of OR asepsis (III).  *NICE (2008):*  • The operating team should wash their hands prior to the first operation on the list using an aqueous antiseptic surgical solution, with a single-use brush or pick for the nails, and ensure that hands and nails are visibly clean (1+, 4).  • Before subsequent operations, hands should be washed using either an alcoholic hand rub or an antiseptic surgical solution (1+).  • If hands are soiled then they should be washed again with an antiseptic surgical solution (4).  *CDC (1999):*  • Keep nails short and do not wear artificial nails (Category IB).  • Perform a preoperative surgical scrub for at least 2 to 5 minutes using an appropriate antiseptic. Scrub the hands and forearms up to the elbows (Category IB).  • After performing the surgical scrub, keep hands up and away from the body (elbows in flexed position) so that water runs from the tips of the fingers toward the elbows. Dry hands with a sterile towel and don a sterile gown and gloves (Category IB).  • Clean underneath each fingernail prior to per- forming the first surgical scrub of the day. Category II  • Do not wear hand or arm jewelry (Category II).  • No recommendation on wearing nail polish (Unresolved Issue).  *ASC SIS (2016):*  • Use of a waterless chlorhexidine scrub is as effective as traditional water scrub and requires less time, but there is no superior agent if used according to manufacturer instructions (WG). |
| 5. Surgical site antiseptic skin asepsis | *Strategies to Prevent SSI (2008):*  • Wash and clean skin around incision site; use an appropriate antiseptic agent (A-II).  *Strategies to Prevent SSI (2014 update):*  • Wash and clean skin around incision site. Use a dual agent skin preparation containing alcohol, unless contraindications exist (I).  *NICE (2008):*  • Prepare the skin at the surgical site immediately before incision using an antiseptic (aqueous or alcohol-based) preparation: povidone-iodine or chlorhexidine are most suitable (1+).  • If diathermy is to be used, ensure that antiseptic skin preparations are dried by evaporation and pooling of alcohol-based preparations is avoided (1+, 1-).  *NICE (2014 update):*  • No further recommendation, current recommendation stands  *CDC (1999):*  • Adhere to principles of asepsis when placing intravascular devices (e.g., central venous catheters), spinal or epidural anesthesia catheters, or when dispensing and administering intravenous drugs (Category IA).  • Handle tissue gently, maintain effective hemostasis, minimize devitalized tissue and foreign bodies (i.e., sutures, charred tissues, necrotic debris), and eradicate dead space at the surgical site (Category IB).  • Use delayed primary skin closure or leave an incision open to heal by second intention if the surgeon considers the surgical site to be heavily contaminated (e.g., Class III and Class IV) (Category IB).  *CDC (2017 update):*  • Perform intraoperative skin preparation with an alcohol-based antiseptic agent unless contraindicated (Category IA–strong recommendation; high-quality evidence).  • Application of a microbial sealant immediately after intraoperative skin preparation is not necessary for the prevention of SSI (Category II–weak recommendation; low-quality evidence suggesting a trade-off between clinical benefits and harms).  • Randomized controlled trial evidence was insufficient to evaluate the trade-offs between the benefits and harms of repeat application of antiseptic agents to the patient’s skin immediately before closing the surgical incision for the prevention of SSI (No recommendation/unresolved issue). |
| 6. Diathermy versus scalpel for surgical incision | *Strategies to Prevent SSI (2008):*  • Handle tissue carefully and eradicate dead space (A-III).  *Strategies to Prevent SSI (2014 update):*  • Handle tissue carefully and eradicate dead space (III).  *NICE (2008):*  • Do not use diathermy for surgical incision to reduce the risk of surgical site infection (1+).  *NICE (2014 update):*  • No further recommendation, current recommendation stands |
| 7. Incisional wound irrigation & intra-cavity lavage | *Strategies to Prevent SSI (2014 update):*  • Perform antiseptic wound lavage (II).  *NICE (2008):*  • Do not use wound irrigation to reduce the risk of surgical site infection (1+, 1-).  • Do not use intracavity lavage to reduce the risk of surgical site infection (1+, 1-).  *WHO (2016):*  • The panel suggests considering the use of irrigation of the incisional wound with an aqueous PVP-I solution before closure for the purpose of preventing SSI, particularly in clean and clean-contaminated wounds (Low).  • The panel suggests that antibiotic incisional wound irrigation should not be used for the purpose of preventing SSI (Low).  • The panel suggests removing the wound drain when clinically indicated. No evidence was found to allow making a recommendation on the optimal timing of wound drain removal for the purpose of preventing SSI (Very low).  *CDC (1999):*  • If drainage is necessary, use a closed suction drain. Place a drain through a separate incision distant from the operative incision. Remove the drain as soon as possible (Category IB).  *CDC (2017 update):*  • Randomized controlled trial evidence suggested uncertain trade-offs between the benefits and harms regarding intraoperative antimicrobial irrigation (eg, intra-abdominal, deep, or subcutaneous tissues) for the prevention of SSI. Other organizations have made recommendations based on the existing evidence, and a summary of these recommendations can be found in the Other Guide- lines section of the narrative summary for this question (No recommendation/unresolved issue).  • Consider intraoperative irrigation of deep or subcutaneous tis- sues with aqueous iodophor solution for the prevention of SSI. Intraperitoneal lavage with aqueous iodophor solution in contaminated or dirty abdominal procedures is not necessary (Category II– weak recommendation; moderate-quality evidence suggesting a trade-off between clinical benefits and harms). |
| 8. Antiseptic/antimicrobial agents prior to wound closure | *NICE (2008):*  • Do not use intraoperative skin re-disinfection or topical cefotaxime in abdominal surgery to reduce the risk of surgical site infection (1+).  *NICE (2014 update):*  • No further recommendation, current recommendation stands  *CDC (2017 update):*  • Do not apply antimicrobial agents (ie, ointments, solutions, or powders) to the surgical incision for the prevention of SSI (Category IB–strong recommendation; low-quality evidence).  • Randomized controlled trial evidence suggested uncertain trade- offs between the benefits and harms regarding antimicrobial dressings applied to surgical incisions after primary closure in the operating room for the prevention of SSI (No recommendation/ unresolved issue). |
| 9. Closure methods |  |
| - Antimicrobial sutures | *Strategies to Prevent SSI (2014 update):*  • Do not routinely use antiseptic-impregnated sutures as a strategy to prevent SSIs (II).  *WHO (2016):*  • The panel suggests the use of triclosan-coated sutures for the purpose of reducing the risk of SSI, independent of the type of surgery (Moderate).  *CDC (2017 update):*  • Consider the use of triclosan-coated sutures for the prevention of SSI (Category II–weak recommendation; moderate-quality evidence suggesting a trade-off between clinical benefits and harms).  *ASC SIS (2016):*  • Triclosan antibacterial suture use is recommended for wound closure in clean and clean-contaminated abdominal cases when available (WG).  *University of Toronto (2017):*   - Antimicrobial-coated sutures may be used to reduce SSIs (Level of evidence: Moderate). |
| - Suture glue |  |
| 10. Selection of wound dressings   - Prophylactic NPWT | *WHO (2016):*  • The panel suggests the use of prophylactic negative pressure wound therapy in adult patients on primarily closed surgical incisions in high-risk wounds for the purpose of the prevention of SSI, while taking resources into account (Low). |
| 11. Room traffic & ventilation | *Strategies to Prevent SSI (2008):*  • Follow American Institute of Architects’ recommendations (C-I).  • Minimise operating room traffic (B-II).  *Strategies to Prevent SSI (2014 update):*  • Follow American Institute of Architects’ recommendations for proper air handling in the OR (III).  • Minimise OR traffic (III).  *NICE (2008):*  • Staff wearing non-sterile theatre wear should keep their movements in and out of the operating area to a minimum (4).  *WHO (2016):*  • The panel suggests that laminar airflow ventilation systems should not be used to reduce the risk of SSI for patients undergoing total arthroplasty surgery (Very low – Low).  *CDC (1999):*  • Maintain positive-pressure ventilation in the operating room with respect to the corridors and adjacent areas (Category IB).  • Maintain a minimum of 15 air changes per hour, of which at least 3 should be fresh air (Category IB).  • Filter all air, recirculated and fresh, through the appropriate filters per the American Institute of Architects’ recommendations (Category IB).  • Introduce all air at the ceiling, and exhaust near the floor (Category IB).  • Do not use UV radiation in the operating room to prevent SSI (Category IB).  • Keep operating room doors closed except as needed for passage of equipment, personnel, and the patient (Category IB).  • Limit the number of personnel entering the operating room to necessary personnel (Category II). |
| 12. Decontamination of: |  |
| - Environment & medical devices | *Strategies to Prevent SSI (2008):*  • Adhere to standard principles of operating room asepsis (A-III).  • Use a US Environmental Protection Agency-approved hospital disinfectant to clean surfaces and equipment (B-III).  *Strategies to Prevent SSI (2014 update):*  • Use an EPA-approved hospital disinfectant to clean visibly soiled or contaminated surfaces and equipment (III).  *CDC (1999):*  • When visible soiling or contamination with blood or other body fluids of surfaces or equipment occurs during an operation, use an EPA-approved hospital disinfectant to clean the affected areas before the next operation (Category IB).  • Do not perform special cleaning or closing of operating rooms after contaminated or dirty operations (Category IB).  • Do not use tacky mats at the entrance to the operating room suite or individual operating rooms for infection control (Category IB).  • Wet vacuum the operating room floor after the last operation of the day or night with an EPA- approved hospital disinfectant (Category II).  • No recommendation on disinfecting environmental surfaces or equipment used in operating rooms between operations in the absence of visible soiling (Unresolved issue).  • Do not perform routine environmental sampling of the operating room. Perform microbiologic sampling of operating room environmental surfaces or air only as part of an epidemiologic investigation (Category IB). |
| - Surgical instruments | *Strategies to Prevent SSI (2008):*  • Sterilize all surgical equipment according to published guidelines; minimise the use of flash sterilisation (B-I).  *Strategies to Prevent SSI (2014 update):*  • Sterilize all surgical equipment according to published guidelines. Minimize the use of immediate-use steam sterilization (II).  *CDC (1999):*  • Sterilize all surgical instruments according to published guidelines (Category IB).  • Perform flash sterilization only for patient care items that will be used immediately (e.g., to reprocess an inadvertently dropped instrument). Do not use flash sterilization for reasons of convenience, as an alternative to purchasing additional instrument sets, or to save time (Category IB).  *ASC SIS (2016):*  • The use of new instruments for closure in colorectal cases is recommended (WG). |
| - Prevention of biofilm | *CDC (2017 update – Ortho):*   - Available evidence suggested uncertain trade-offs between the benefits and harms regarding cement modifications and the prevention of biofilm formation or SSI in prosthetic joint arthroplasty. (No recommendation/unresolved issue.) - The search did not identify studies evaluating prosthesis modifications for the prevention of biofilm formation or SSI in prosthetic joint arthroplasty. (No recommendation/unresolved issue.) - The search did not identify studies evaluating vaccines for the prevention of biofilm formation or SSI in prosthetic joint arthroplasty. (No recommendation/unresolved issue.) - The search did not identify studies evaluating biofilm control agents, such as biofilm dispersants, quorum sensing inhibitors, or novel antimicrobial agents, for the prevention of biofilm formation or SSI in prosthetic joint arthroplasty. (No recommendation/unresolved issue.) |
| **POST-OPERATIVE PHASE** | |
| 1. Surgical antibiotic prophylaxis prolongation | *Strategies to Prevent SSI (2008):*  • Stop prophylaxis within 24 hours after the procedure for all procedures expect cardiac surgery; for cardiac surgery, antimicrobial prophylaxis should be stopped within 48-hours (A-I).  *Strategies to Prevent SSI 2014 update):*  • Stop agent within 24 hours after the procedure for all procedures (II).  *WHO (2016):*  • The panel recommends against the prolongation of SAP after completion of the operation for the purpose of preventing SSI (Moderate).  *CDC (2017 update):*  • In clean and clean-contaminated procedures, do not administer additional prophylactic antimicrobial agent doses after the surgical incision is closed in the operating room, even in the presence of a drain (Category IA–strong recommendation; high-quality evidence).   - (Ortho) In prosthetic joint arthroplasty, recommendation 1E applies: in clean and clean-contaminated procedures, do not administer additional antimicrobial prophylaxis doses after the surgical incision is closed in the operating room, even in the presence of a drain. (Category IA–strong recommendation; high-quality evidence.)   *ASC SIS (2016):*  • There is no evidence that prophylactic antibiotic administration after incision closure decreases SSI risk; prophylactic antibiotics should be discontinued at time of incision closure (exceptions include implant- based breast reconstruction, joint arthroplasty, and cardiac procedures where optimal duration of antibiotic therapy remains unknown) (WG).  *University of Toronto (2017):*   - Antibiotics should not be given postoperatively unless there is an indication other than for prophylaxis (Level of evidence: High) - Patients who have indwelling drains or intravascular catheters do not require additional prophylaxis (Level of evidence: Moderate) |
| 2. Timing of dressing changes | *CDC (1999):*  • Protect with a sterile dressing for 24 to 48 hours postoperatively an incision that has been closed primarily (Category IB).  - No recommendation to cover an incision closed primarily beyond 48 hours, nor on the appropriate time to shower or bathe with an uncovered incision (Unresolved Issue).  *ASC SIS (2016):*  • There is no evidence in the literature that timing of dressing removal increases SSI risk (WG). |
| 3. Postoperative wound cleansing | *NICE (2008):*  • Use sterile saline for wound cleansing up to 48 hours after surgery (4).  • Advise patients that they may shower safely 48 hours after surgery (1+).  • Use tap water for wound cleansing after 48 hours if the surgical wound has separated or has been surgically opened to drain pus (4).  *ASC SIS (2016):*  • Early showering (12 hours postoperative) does not increase the risk of SSI (WG). |
| 4. Topical antimicrobial agents for wound healing by primary intention | *NICE (2008):*  • Do not use topical antimicrobial agents for surgical wounds that are healing by primary intention to reduce the risk of surgical site infection (1+).  *ASC SIS (2016):*  • Topical antibiotics can reduce SSI for specific cases, including spine surgery, total joint arthroplasty, and cataract surgery, but there is insufficient evidence to recommend routine use at this time (WG).  • Mupirocin topic antibiotic application can decrease SSI compared with a standard dressing (WG). |
| 5. Dressings for wound healing by secondary intention | *NICE (2008):*  • Do not use Eusol and gauze, or moist cotton gauze or mercuric antiseptic solutions to manage surgical wounds that are healing by secondary intention (1-).  • Use an appropriate interactive dressing to manage surgical wounds that are healing by secondary intention (1-). |
| 6. Wound debridement | *NICE (2008):*  • Do not use Eusol and gauze, or dextranomer or enzymatic treatments for debridement in the management of surgical site infection (1-). |
| 7. Use of advanced dressings | *WHO (2016):*  • The panel suggests not using any type of advanced dressing over a standard dressing on primarily closed surgical wounds for the purpose of preventing SSI (Low). |
| 8. Wound related analgesia | No recommendations made. |
| 9. Specialist wound care services | *NICE (2008):*  • Refer to a tissue viability nurse (or another healthcare professional with tissue viability expertise) for advice on appropriate dressings for the management of surgical wounds that are healing by secondary intention (4).  • Although there is no direct evidence to support the provision of specialist wound care services for managing difficult to heal surgical wounds, a structured approach to care (including preoperative assessments to identify individuals with potential wound healing problems) is required in order to improve overall management of surgical wounds. To support this, enhanced  education of healthcare workers, patients and carers, and sharing of clinical expertise will be required (4). |
| 10. Antibiotic treatment of SSI & treatment failure | *NICE (2008):*  • When surgical site infection is suspected (i.e. cellulitis), either *de novo* or because of treatment failure, give the patient an antibiotic that covers the likely causative organisms. Consider local resistance patterns and the results of microbiological tests in choosing an antibiotic (4). |
| **DOCUMENTATION** | |
| 1. Surgical wound classification | *CDC (1999):*  • Use CDC definitions of SSI without modification for identifying SSI among surgical inpatients and outpatients (Category IB).  • Assign the surgical wound classification upon completion of an operation. A surgical team member should make the assignment (Category II). |
| 2. Record variables associated with increased SSI risk | *CDC (1999):*  • For each patient undergoing an operation chosen for surveillance, record those variables shown to be associated with increased SSI risk (e.g., surgical wound class, ASA class, and duration of operation) (Category IB). |
| 3. Calculation of operation-specific SSI rates | *Strategies to Prevent SSI (2008):*  • Provide ongoing feedback on SSI surveillance and process measures to surgical and perioperative personnel and leadership (A-II).  *Strategies to Prevent SSI (2014):*  • Perform surveillance for SSI (II).  • Perform an SSI risk assessment (III).  *CDC (1999):*  • Periodically calculate operation-specific SSI rates stratified by variables shown to be associated with increased SSI risk (e.g., NNIS risk index) (Category IB). |
| 4. Report SSI rates to surgical team members | *Strategies to Prevent SSI (2008):*  • Provide ongoing feedback on SSI surveillance and process measures to surgical and perioperative personnel and leadership (A-II).  *Strategies to Prevent SSI (2014):*  • Provide ongoing feedback of SSI rates to surgical and perioperative personnel and leadership (II).  *CDC (1999):*  • Report appropriately stratified, operation-specific SSI rates to surgical team members. The optimum frequency and format for such rate computations will be determined by stratified case-load sizes (denominators) and the objectives of local, continuous quality improvement initiatives (Category IB). |
| **PATIENT/FAMILY EDUCATION** | |
| 1. Incision care/management | *NICE (2008):*  • Always inform patients after their operation if they have been given antibiotics (NR).  *CDC (1999):*  • Educate the patient and family regarding proper incision care, symptoms of SSI, and the need to report such symptoms (Category II). |
| 2. Symptoms/ recognising/ reporting SSI | *Strategies to Prevent SSI (2008):*  • Educate patients and their families about SSI prevention as appropriate (A-III).  • Educate patients and their families about SSI prevention as appropriate (III).  *NICE (2008):*  • Offer patients and carers clear, consistent information and advice throughout all stages of their care. This should include the risks of surgical site infections, what is being done to reduce them and how they are managed (NR).  • Offer patients and carers information and advice about how to recognise a surgical site infection and who to contact if they are concerned. Use an integrated care pathway for healthcare associated infections to help communicate this information to both patients and all those involved in their care after discharge (NR).  *CDC (1999):*  • Educate the patient and family regarding proper incision care, symptoms of SSI, and the need to report such symptoms (Category II). |
| 3. Smoking cessation | *Strategies to Prevent SSI (2008):*  • Encourage smoking cessation within 30 days before surgery (A-II).  *Strategies to Prevent SSI (2014):*  • Encourage smoking cessation within 30 days before surgery (I).  *CDC (1999):*  • Encourage tobacco cessation. At minimum, instruct patients to abstain for at least 30 days before elective operation from smoking cigarettes, cigars, pipes, or any other form of tobacco consumption (e.g., chewing/dipping) (Category IB).  *ASC SIS (2016):*  • Smoking cessation 4 to 6 weeks before surgery reduces SSI and is recommended for all current smokers, especially those undergoing procedures with implanted materials. There is no literature to support cessation of marijuana and electronic cigarette use to prevent SSI, but cessation is recommended before surgery based on expert consensus (WG).  • American College of Surgeons patient education materials support the use of nicotine lozenges, nicotine gum, and medication to aid in smoking cessation (WG). |
| 4. Discharge planning | *NICE (2008):*  • Offer patients and carers information and advice on how to care for their wound after discharge (1+). |
